# 

**Published:** 1839-01

**Authors:** 


### CONTENTS OF No. XIII.

OF THE

### British and Foreign Medical Review.

JANUARY, 1839.

---

### Part First.—Analytical and Critical Reviews.

PAGE

Art. I.—1. A Treatise on Insanity, and other Disorders affecting the Mind. By James Cowles Prichard, m.d. f.r.s. &c. . . . . 1

2. Des Maladies Mentales considérées sous les Rapports Médical, Hygiènique, et Médico-légal. Par E. Esquirol . . . . . ib.
Mental Maladies considered in relation to Medicine, Hygiène, and Medical Jurisprudence. By E. Esquirol

3. Essay on the Classification of the Insane. By M. Allen, m.d. . . ib.

4. A Treatise on the Nature, Symptoms, Causes, and Treatment of Insanity; with Practical Observations on Lunatic Asylums, and a Description of the Pauper Lunatic Asylum for the County of Middlesex, at Hanwell, with a detailed Account of its Management. By Sir W. C. Ellis, m.d. . . ib.

5. Delle Malattie della Mente, ovvero delle diverse Specie di Follie. Opera di Luigi Ferrarese, Dottor di Medicina, &c. . . . ib.
Of the Maladies of the Mind, or the different Species of Madness. By Luigi Ferrarese, m.d. &c.

6. Tenth Report of the Directors of the Connecticut Retreat for the Insane. 1834 ib.

7. Report of the Lunatic Asylum in St. Petersburgh. 1834. . . ib.

8. Saggio sulla Statistica Medica della Real Casa dei Matti di Palermo. Scritto da Antonio Greco, Medico ordinario, &c. . . . . .
Medical Statistics of the Royal Institution for the Insane at Palermo. By A. Greco, Physician in ordinary, &c.

9. State of the New-York Hospital, and Bloomingdale Asylum. 1835 and 1836. ib.

10. Sixteenth and Eighteenth Report of the Directors of the Dundee Lunatic Asylum. 1836 and 1838 . . . . . . ib.

11. State of an Institution near York, called The Retreat, for Persons afflicted with Disorders of the Mind. 1836 . . . . . ib.

12. Reports and other Documents relating to the State Lunatic Hospital at Wores ter, Mass. 1837 . . . . . . ib.

13. The Forty-fourth Report of the Visiting Justices, and the Seventh Report of the Resident Physician and Treasurer of the Middlesex Pauper Lunatic Asylum ib.

14. Physician's Report to the Managers of the Lunatic Asylum of Aberdeen. 1838 ib.

15. On the Statistics of English Lunatic Asylums, and the Reform of their Public Management. By William Farr . . . . ib.

16. Observations on the Management of Madhouses; illustrated by Occurrences in the West-Riding and Middlesex Asylums. By Caleb Crowther, m.d. . ib.

Art. II.—1. Traité théorique et pratique de la Derivation contre les Affections les plus communes en général, telle que la Pléthore, l'Inflammation, l'Hémorragie, &c. Par L. F. Gondret, m.d. . . . . . 56
Theoretical and Practical Treatise on Derivation as a Remedy for some of the most common Affections, such as Plethora, Inflammation, Hemorrhage, &c. By L. F. Gondret, m.d.

2. Counter-Irritation, its Principles and Practice, illustrated by one hundred Cases of the most painful and important Diseases effectually cured by external Applications. By A. B. Granville, m.d. f.r.s. . . . ib.

3. Counter-Action used as a Means of Cure, with Remarks on the Uses of the Issue. By John Epps, m.d. . . . ib.

PAGE

ART. III.—1. Clinique des Maladies des Enfans Nouveau-nés. Par F. L. VALLEIX 74
Clinical Observations on the Diseases of New-born Infants. By Dr. VALLEIX.
De Tumore Cranii recens Natorum sanguineo Symbolæ. Quibus, viro experientissimo ELIÆ HERSCHEL, M.D. &c., gratulatur J. A. BURCHARD, M.D. . ib.
Contributions on the subject of the Sanguineous Tumour of the Skull of New-born Infants. By J. A. BURCHARD, M.D.

ART. IV.—1. Die Influenza. Ein historischer und ätiologischer Versuch. Von HEINRICH SCHWEICH, Dr. der Med. und Chir. . . . 95
An historical and ætiological Essay on the Influenza. By Dr. H. SCHWEICH.
2. Die Influenza oder Grippe, nach den Quellen historisch-pathologisch dargestellt. Von Dr. GOTTLIEB GLUGE . . . . . ib.
An historical and practical Treatise on Influenza. By Dr. GLUGE.
3. A Treatise on the Influenza of 1837. By PEYTON BLAKISTON, M.D. . ib.
4. Report upon the Influenza, or Epidemic Catarrh of the Winter 1836-7. By ROBERT J. N. STREETEN. M.D. . . . . . . ib.

ART. V.—The Stomach in its Morbid States: being a Practical Enquiry into the Nature and Treatment of Diseases of that Organ. By L. PARKER, M.R.C.S. 115

ART. VI.—Die Zurechnungsfähigkeit der Schwangern und Gebärenden beleuchtet Von Dr. JOHANN. CHR. GOETFR. JÖRG . . . . 129
On the Legal Responsibility of Females during Pregnancy and Parturition. By Dr. J. C. G. JÖRG.

ART. VII.—Medico-Chirurgical Transactions, published by the Royal Medical and Chirurgical Society of London. Vol. XXI. . . . 140
Mr. GULLIVER on Necrosis . . . . . ib.
Mr. C. HUTCHINSON on the Freedom of Sailors from Calculous Diseases . 141
Dr. BOSTOCK on the Examination of Urine . . . . 142
Dr. CLENDINNING on the Condition of the Vital Organs and Viscera in Disease ib.
Mr. HAWKINS on Malignant Diseases of the Skin of the Face . . 143
Mr. MALCOLMSON on a peculiar Symptom in some Cases of enlarged Liver . 146
Dr. WILSON on Nervous Affections peculiar to young Women . . ib.
Dr. MOORE on the secondary Occurrence of Measles in a Child . . 147
Mr. TRAVERS on the Removal of a Clavicle with a Tumour situated in it . ib.
Mr. ANCELL'S Case of universal Purulent Deposition into the Joints . 149.
Dr. BUDD and Mr. BUSK'S Cases of Malignant Cholera . . . ib.
Mr. THURNAM on Aneurisms of the Heart . . . . ib.
Dr. LEE on a Female who has four Mammæ and Nipples . . 151
Dr. WILSON on the Results of Poisoning by Sulphuric Acid . . ib.
Mr. HUNT on the Use of Arsenic in some Affections of the Uterus . ib.
Mr. PERRY'S Case of Excision of the entire Lower Jaw . . . 152
Dr. BUDD on Concentric Hypertrophy of the Heart . . . 153
Mr. HAWDEN'S Case of Popliteal Aneurism . . . . ib.
Mr. TYRRELL on Inflammation of the Conjunctiva, &c. &c. . . 155

ART. VIII.—Urinary Diseases and their Treatment. By ROBERT WILLIS, M.D. . 157

ART. IX.—Principles of General and Comparative Physiology, intended as an Introduction to the Study of Human Physiology, and as a Guide to the Philosophical Pursuit of Natural History. By WM. B. CARPENTER, M.R.C.S. . 167

ART. X.—1. Historisch-kritisch Darstellung der Pockenseuchen, des gesammten Impf-und Revaccinationswesens in Königreiche Würtemberg, innerhalb die fünf Jahre, Juli, 1831, bis Juni, 1836. Von Prof. Dr. FRANZ HEIM . 186
Historical and Critical View of the Small-pox Epidemics, &c. By Dr. HEIM.
2. Resultat der Revaccination in der Koniglich Würtembergischen Militär, in den Jahren 1833, 1834, und 1835. Von Professor HEIM . . . ib.
Results of the Revaccination of the Royal Wurtemberg Army. By Dr. HEIM.

ART. XI.—A Treatise on the Nature and Treatment of the Hooping-cough, and its Complications, illustrated by Cases. By G. H. ROE, M.D. . . 210

ART. XII.—Elements of Physiology. By THOMAS J. AITKIN, M.D. . . 214

2. Popular Physiology. By PERCEVAL B. LORD, M.B. . . . ib.

3. Outlines of Human Physiology. By GEORGE HAYWARD, M.D. . . ib.

ART. XIII—Ophthalmia. By J. SLADE, M.D. . . . . 217

### Part Second.—Bibliographical Notices.

PAGE

Art. I.—A Treatise on Inflammation. By James Macartney, m.d. . . 221

Art. II.—On Kephalosis. By John Gardner, Surgeon . . . 222

Art. III.—Chatham Army Medical Museum Reports . . . 224

Art. IV.—Pettigrew's Medical Portrait Gallery . . . . 225

Art. V.—The Power, Wisdom, and Goodness of God. By G. Burnett, m.r.c.s. 227

Art. VI.—Dr. Waugh on the Cerebro-spinal Phenomena . . . 228

Art. VII.—Human Physiology. By Robley Dunglison, m.d. . . 229

Art VIII.—De Auscultatione Obstetricia. Auct. A. C. Conradi . . 230

On Obstetrical Auscultation. By A. C. Conradi, m.d.

Art. IX.—A System of Practical Surgery. By John Lizars. Part I. . 231

Art. X.—Questiones Emmenologicas, scripsit Joh. Fried. Krieg, m.d. . 233

Emmenological Questions. By John Frederick Krieg, m.d.

Art. XI.—Practical Surgery. By Robert Liston. Second Edition . . 235

Art. XII.—De Salivâ ejusque vi et utilitate. Auct. G. Van Setten . . 236

On the Action and Uses of the Saliva. By G. Van Setten, m.d.

---

### Part Third.—Selections from the British and Foreign Journals.

### I. THE FOREIGN JOURNALS.

#### ANATOMY AND PHYSIOLOGY.

Pr. Volkmann on the Structure and Functions of the Cerebral Nerves of the Frog 237
Flourens on the Comparative Structure of the Cutaneous and Mucous Membrane 239
M. Bouvier on the Cicatrization of Tendons . . . . . 240
Prof. Sebastian on the Venous Circle of the Mammary Areola . . ib.
on accessory Supra-renal Capsules . . . 241

#### PATHOLOGY, PRACTICAL MEDICINE, AND THERAPEUTICS.

M. Cruveilhier on simple Chronic Ulcer of the Stomach . . . ib.
M. Pratbernon on the Miliary or Sweating Fever (Suette Miliaire) . . 244
Dr. Von Haselberg on Hydriodate of Potash in secondary Syphilis, Scrofula, &c. 245
Dr. Heidler's Cases of Facial Paralysis . . . . . . 247
M. Monat on a Canal filled with Serum in the centre of the Spinal Cord . 248
Pr. Sebastian on the Resistance of the Peritoneum to Perforation of the Intestine . ib.
Dr. Ollivier on a Gaseous Fluid in the Circulation as a Cause of sudden Death . 249
Dr. Tonelli's Case of Congenital Transposition of the Viscera . . ib.

#### SURGERY.

Prof. Dieffenbach on the Removal of the Bones of the Face . . 250
MM. Breschet and Guyot on the Cicatrization of Wounds . . . 251
M. Stanski's Case of a Spike of Oat introduced into the Bronchi . . ib.
M. Blandin's New Theory and Treatment of Erysipelas . . . 252
Dr. Hatin's New Mode of tying Polypi of the Pharynx . . . 253
Dr. Heil on Recovery from a Wound of the ascending Aorta . . 254
M. Velpeau's Treatment of Varicose Veins . . . . ib.
Dr. Fricke on the immoveable Fracture-bandage . . . . ib.
Deglutition of a large Silver Coin . . . . . . 255

#### MIDWIFERY.

Dr. Villeneuve's Memoir on Absorption of the Placenta . . . ib.
Dr. Galiay on Extra-uterine Pregnancy occurring twice in the same Patient . 257

#### MEDICAL JURISPRUDENCE.

Dr. Graff's Case of suspected Infanticide . . . . ib.
Dr. Speyer on a Fatal Wound of the Head . . . . 259
Dr. Carganico's Case of Suicidal Strangulation . . . . 261

#### MEDICAL STATISTICS.

Salvatore de Renzi's View of the Operations for Lithotomy, and their Results, in the Hospital of Santa Maria di Loreto in Naples . . . 262

### II. THE AMERICAN AND COLONIAL JOURNALS.

PAGE

PHYSIOLOGY.

Dr. Hooker on the Relation between the Respiratory and Circulating Functions . 263

MEDICINE.

Dr. Fisher's Observations on Cerebral Auscultation . . . 265
Dr. Ware on the Treatment of Delirium Tremens . . . 268

SURGERY.

Dr. Maxwell's Treatment of Hydrophobia on Physiological Principles . 270

MEDICAL STATISTICS.

Mortality Table for the City of New York (with the Names of the Diseases), for a Period of thirty-two years. By H. G. Dunnel, M.D. . . 272

### III. THE BRITISH JOURNALS.

ANATOMY AND PHYSIOLOGY.

Dr. Corrigan on the Mechanism of Bruit de Soufflet, &c. . . . 276
Mr. Tomes on the Structure of the Teeth, &c. . . . . ib.
Mr. Hilton on the Decussation of Fibres at the Junction of the Medulla Spinalis 277
Mr. Eagle on the Causes, Nature, and Prevention of Small-pox . . 278
Mr. Whitehouse's New Casts for Anatomical Specimens . . . ib.
Dr. Davy's Experiments on the Blood in connexion with the Theory of Respiration 279
Mr. Wheatstone on the Phenomena of Binocular Vision . . . ib.
Dr. Wallace's Cases of Muscæ Volitantes . . . . 280

PATHOLOGY, PRACTICAL MEDICINE, AND THERAPEUTICS.

Dr. Kennion on the Origin of the Optic Nerves . . . 282
Mr. Laycock on irregular and aggravated Forms of Hysteria . . . ib.
Dr. Lombard's Remarks on the Hooping-cough . . . . 283
Dr. Bigsby on the use of Oil in Colica Pictonum . . . . 284
Dr. Simpson's Cases of Peritonitis in the Fœtus in Utero . . . ib.

SURGERY.

Mr. Fergusson on Lithotrity . . . . . . 286
Dr. Greenhow on a new Method of treating Burns . . . 287
Mr. Coley on Vesico-vaginal Fistula cured by a new Mode of Operating . 289
Mr. Ranking's Case of Strangulated Mesenteric Hernia . . . 290
Mr. Wigan on a simple Mode of dilating the Urethra . . . ib.

MIDWIFERY.

Dr. Churchill on the Umbilical Cord in the Human Subject, (Prolapsus) . 291

TOXICOLOGY.

Mr. Turner on the Poisonous Effects of the Substance used for what is termed Printing "in Gold" . . . . . . . ib.

MATERIA MEDICA.

Dr. Granville's Counter-irritant Lotions . . . . 292

MEDICAL STATISTICS.

Dr. Green on the Mortality and Frequency of Disease amongst Children . 294

Meteorological Results at Chiswick . . . . . . ib.

### Part Fourth.—Medical Intelligence.

Dr. Baron on the Identity of Small-pox and Cow-pox . . . 295
OBITUARY: Dr. Broussais, Dr. Hedenus, Dr. Bluff . 297, 298
Books received for Review . . . . . 299

